# Current management approach to hidradenocarcinoma: a comprehensive review of the literature

**DOI:** 10.3332/ecancer.2015.517

**Published:** 2015-03-19

**Authors:** Abhishek Soni, Nupur Bansal, Vivek Kaushal, Ashok Kr Chauhan

**Affiliations:** Department of Radiotherapy, Pt. B. D. Sharma Post Graduate Institute of Medical Sciences, Rohtak, Haryana, India

**Keywords:** hidradenocarcinoma, management, wide local excision, radiotherapy

## Abstract

Hidradenocarcinoma is a rare malignant adnexal tumour which arises from the intradermal duct of eccrine sweat glands. The head and neck are the most common sites of hidradenocarcinoma, but rarely it can occur on the extremities. As it is an aggressive tumour, regional lymph nodes and distant viscera are the most common sites of metastasis. Diagnosis is confirmed by histopathology and immunohistochemistry. Hidradenocarcinoma should be differentiated from benign and malignant adnexal tumours. Being an aggressive and rare tumour, no uniform treatment guidelines have been documented so far for metastatic hidradenocarcinoma. Wide local excision is the mainstay of the treatment, but because of high local recurrence, radiotherapy in a dose of 50Gy–70Gy and/or 5-fluorouracil and capecitabine-based combination chemotherapy may be given to further improve local control. Other treatment strategies are targeted therapies like trastuzumab, EGFR inhibitors, PI3K/Akt/mTOR pathway inhibitors, hormonal agents like antiandrogens, electrochemotherapy, or clinical trials.

## Introduction

Hidradenocarcinoma is a rare malignant tumour arising from the sweat glands and accounts for less than 0.001% of all tumours [1, 2, 3]. Based on histochemical studies and electron microscopy [4], hidradenocarcinoma is also often referred to as clear cell eccrine carcinoma, malignant nodular hidradenoma, malignant clear cell acrospiroma, malignant clear cell hidradenoma, or primary mucoepidermoid cutaneous carcinoma [5]. It arises conventionally *de novo* and rarely results from a pre-existing hidradenoma [3, 5]. This rare malignancy presents initially and/or most frequently on the head and neck region especially on the face [6, 7]. The diagnosis is based on histological and immunohistochemical analysis [8, 9]. Hidradenocarcinoma should be differentiated from several benign tumours like hemangiomas, lipomas, and lymphangiomas; and malignant tumours like malignant melanoma, basal cell carcinomas, squamous cell carcinomas, and [10] other malignant adnexal carcinomas [10]. There is no consensus treatment for hidradenocarcinoma. Surgery is the mainstay of the treatment and postsurgical survival rate at five years is found to be less than 30% [11]. The postsurgical recurrence rate is 50% [12]. So, for improving local control, external beam radiotherapy may be given as an adjuvant treatment, but it is controversial [13]. Systemic chemotherapy has an interesting role in the metastatic setting [13]. When radiotherapy (RT) is used, high doses ranging from 50 Gy–70 Gy are usually necessary [14]. First line chemotherapeutic agents include 5-fluorouracil-based regimen [7] and capecitabine; [7, 15] and second line agents include doxorubicin, platins, [7] cyclophosphamide, vincristine, and bleomycin [14]. The current focus for treatment of hidradenocarcinoma is on targeted therapies like trastuzumab, EGFR (epidermal growth factor receptor) inhibitors, PI3K/Akt/mTOR pathway inhibitors, hormonal agents like antiandrogens and electrochemotherapy [16] but more future trials are required. As there are no established treatment guidelines for hidradenocarcinoma, one of the objectives of this article is the in-depth review of current treatment options, their indications, advantages and disadvantages, and to see the recurrence and survival of each treatment modality in hidradenocarcinoma.

## Methods

This literature review was performed by conducting a systematic search of PUBMED, MEDLINE and PMC, including all articles up to November 2014. Keywords used for the search included ‘hidradenocarcinoma’, ‘malignant clear cell acrospiroma’, ‘malignant nodular hidradenoma’, ‘clear cell eccrine carcinoma’, ‘malignant clear cell hidradenoma’ or ‘primary mucoepidermoid cutaneous carcinoma’. All articles were reviewed and were included if they were relevant to the topic, and deemed to be of good quality. The references for each article were reviewed to identify further articles of relevance.

## Discussion

### Epidemiology

Hidradenocarcinoma is a rare, aggressive, intradermal, skin adnexal malignant tumour of the sweat glands [10, 17]. Sweat gland carcinoma is a rare malignancy and the United States reported an incidence of around 0.05% [17]. Primary eccrine carcinomas are rare tumours and make up less than 0.01% of all skin cancers [18, 19]. Hidradenocarcinoma accounts for approximately 6% of malignant eccrine tumours [20] and accounts for less than 0.001% of all tumours [1, 2]. In 1865, French pathologist Victor Andre Cornil reported first case of sweat gland carcinoma [21]. The fifth to the seventh decade of life is the most common age group of presentation of hidradenocarcinoma [17] with the mean age of onset being 50 years [8]. Hidradenocarcinoma presents with a slight female predominance [8] and without any racial prevalence [17]. It arises conventionally *de novo* and rarely results from a pre-existing hidradenoma [3, 5].

### Pathology

**Figure 1.**
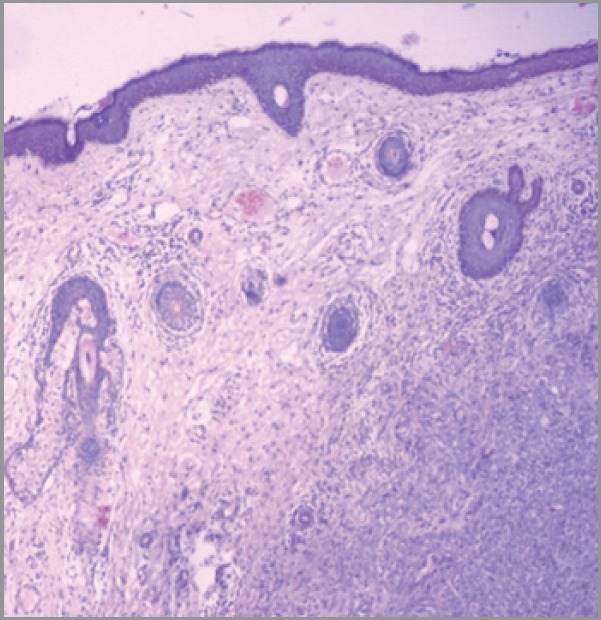
A hematoxylin and eosin-stained photomicrograph (40X) of hidradenocarcinoma showing skin lined with stratified squamous epithelium. The dermis shows lobulated mass extending into subcutaneous tissue revealing follicular and clear cells.

**Figure 2.**
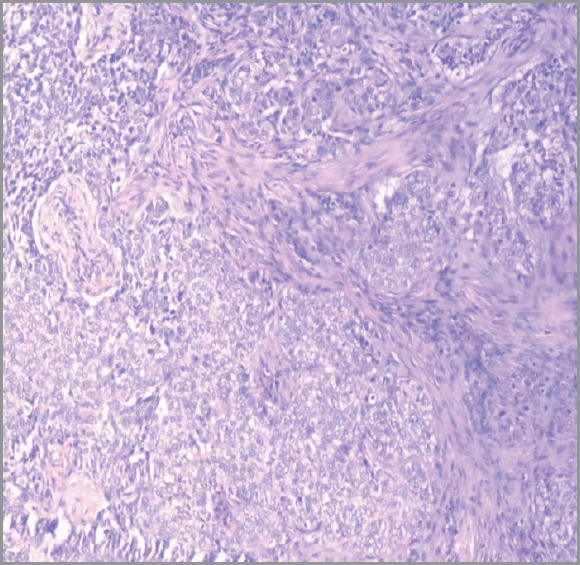
A hematoxylin-eosin staining of hidradenocarcinoma showing polygonal cells with rounded nucleus and slightly basophilic cytoplasm clear cells with rounded nucleus and clear cytoplasm (200X).

Hidradenocarcinomas are seen in 1 in 13,000 dermatopathology biopsies [20]. On gross pathological examination, hidradenocarcinomas typically present as well-circumscribed nodules over the superficial skin [10]. On histopathological examination, hidradenocarcinoma has two different cell types: eosinophilic cytoplasm laiden darker fusiform/spindle cells and larger clear cells exhibiting atypical mitotic figures and nuclear pleomorphism [10]. Clear cells are round and rich in glycogen and express PAS (periodic acid-Schiff), whereas spindle cells are present at the periphery of the lobules. As in other eccrine tumours, hidradenocarcinoma cells express cytokeratins, EMA (epithelial membrane antigen), CEA (carcinoembryonic antigen) and S100 protein [14]. Apocrine differentiation is frequently seen. Remnants of nodular hidradenoma may also be seen. An infiltrative growth pattern is not universally seen [5]. Rarely, ductal structures and cystic spaces are seen. Other criteria may be seen such as vascular invasion, deep extension, necrosis, or perineural invasion [22]. Histologically, hidradenocarcinoma is difficult to differentiate from hidradenoma, a benign tumour [10]. To differentiate these two entities, histological criteria is used including angiolymphatic invasion into surrounding tissue, mitoses in clear cells, greater mitotic activity, and loss of circumscription [2, 23]. Presence of tumour cords with peripheral invasion may be used as a sole criteria for the diagnosis of hidradenocarcinoma, so absence of other criteria does not exclude diagnosis of hidradenocarcinoma [7]. Figures 1 and 2 show the histological features of hidradenocarcinoma.

#### Immunohistochemical analysis

On immunohistochemistry, hidradenocarcinomas are variably positive for androgen receptor (AR), estrogen receptor (ER), progesterone receptor (PR), EGFR (epidermal growth factor receptor), and HER-2 (human epidermal growth factor receptor 2) [24]. AR, PR, ER, HER-2, and EGFR expression was seen in 36%, 16%, 27%, 12% and 85%, respectively. Trisomy or polysomy of EGFR was detected by FISH in 30%. Mutations of PIK3CA, AKT-1, and TP53 were detected in 23% cases [14]. Anti-androgen therapy may be used in apocrine carcinoma subtype because of strong correlation of AR expression in these type of cancers. In skin adnexal carcinomas, it was suggested that molecular mechanisms excluding gene amplification may have a role in EGFR overexpression because of lack of correlation between the polysomy/ gene amplification, and protein expression [24].

Table 1 shows the classification of cutaneous sweat gland lesions [2] which is very complex as these lesions have a broad spectrum of histology, and exact origin and pathogenesis of many lesions is still under investigation and not clear [5, 25, 26].

### Natural history

The tumour or lesion may expand locally for a quite variable period of time, which may last from months to years to decades. Generally, most patients remain asymptomatic. The patient may present with discomfort, pain, ulceration, or bleeding upon physical contact. At some point in time, the tumour may demonstrate an aggressive clinical course with regional extension or distant metastatic spread, usually to the lymph nodes. The mechanism for this type of spread is not clear yet. Surprisingly, the patient may remain asymptomatic even after metastatic spread of the disease [10]. Nodal involvement usually precedes visceral metastasis that occurs in 39% and 28% of patients, respectively [14].

### Clinical features

This rare malignancy presents initially and/or most frequently on the head and neck region especially on the face and later and/or rarely on the distal extremities [6, 7, 8]. It has been reported rarely on the trunk, abdomen, and groin and even more rarely on the digits, elbows, and scalp [2]. Usually, the tumour presents as a solitary, subcutaneous, firm nodule [27] or erythematous plaque [6, 7] with telangiectasia and/ or ulceration [14] which mimics ‘benign’ solitary skin lesion, and it may slowly expand circumferentially, or it may maintain a stable size of 1–5 cm [2]. It may appear fleshy red, gray, or violet with normal overlying skin [6, 7].

### Diagnosis and workup

The diagnosis is based on histological and immunohistochemical analysis of the biopsy material [8, 9]. x-ray, ultrasonography, computed tomography scans, magnetic resonance imaging, and positron emission tomography scans are used to rule out local or distant spread of the primary tumour [9]. Hidradenocarcinoma arise from the intradermal duct of eccrine sweat glands [28]. Sweat glands can be either apocrine or eccrine in nature histologically. Eccrine glands are found all over the skin but are mostly seen over axillae, palms, and soles. Apocrine glands are present mainly around the nipples, in the axillae, anogenital region, chest, and abdomen [29].

#### Differential diagnosis

Hidradenocarcinoma can be mistaken clinically with infundibular and pilar cysts, cutaneous tuberculosis or dermatofibrosarcoma protuberans [6, 7]. Differential diagnosis of hidradenocarcinoma includes benign tumours like hemangiomas, lipomas, lymphangiomas; and malignant tumours like basal cell carcinomas, squamous cell carcinomas, malignant melanoma [10] other malignant adnexal carcinomas such as eccrine adenocarcinomas, adenoid cystic eccrine carcinomas, mucinous eccrine carcinomas, aggressive digital papillary adenocarcinomas, and metastatic tumours to the skin. Hidradenocarcinoma may also resemble primary carcinomas from the breast, salivary glands, and lungs [10]. Hidradenocarcinoma and clear cell hidradenoma may also mimic metastatic clear cell carcinomas including thyroid, lungs, or renal cell carcinomas. However, the first two tumours are generally differentiated by their positivity to TTF-1 (thyroid transcription factor-1), and the latter by the presence of focal granular necrosis and haemorrhage within the lesion, and its prominent vascularity. Renal cell carcinoma also expresses CD10 and epithelial membrane antigen (EMA) [5]. Other differentials include basaloid eccrine carcinoma, eccrine ductal carcinoma, clear cell eccrine carcinoma, and other non-specified sweat gland carcinomas. These tumours have prominent eccrine component/differentiation, no characteristic clinical picture, and are usually very difficult to differentiate from metastatic carcinomas. They generally lack an epidermal connection and simulate carcinomas from other parts of the body, including breast, thyroid, salivary glands, and renal cell carcinomas. Eccrine ductal carcinoma resembles breast ductal carcinomas. The differential diagnosis of basaloid eccrine carcinoma is wide, and includes Merkel cell carcinoma, Ewing’s sarcoma, metastatic carcinoma, as well as small cell melanoma, and squamous cell carcinoma [5].

### Staging

TNM classification and staging system is followed for hidradenocarcinoma as depicted in Table 2 [30].

### Treatment

As hidradenocarcinoma is an aggressive and extremely rare tumour, there is no consensus treatment for it till to date [7]. The treatment of choice for hidradenocarcinomas is surgery in the form of wide local excision [31, 32]. So early diagnosis is critical to treatment outcome and quality of life for patients [10]. The primary surgical treatment involves wide local excision with or without lymph node dissection [33, 34]. Radiation therapy is used selectively in patients, and since chemotherapy effectiveness has not been proven yet, it is not used extensively [35].

#### Surgery

Surgery is the mainstay of treatment, consisting of wide local excision with negative margins. [13, 36, 37]. Classical carcinoma surgery may be used but two-step surgery may enhance the quality of the margin control and excellent control can be achieved by Mohs micrographic surgery [38]. Table 3 shows that at least a 3 cm margin should be taken for hidradenocarcinoma, but if these large margins cannot be respected because of anatomical or functional conditions, a strict histological examination of lateral margins by the pathologist is mandatory. In these cases, a two-step surgery or Mohs micrographic surgery can be very helpful in local control of the disease [14].

Once initial diagnosis is made, wide surgical excision should be performed as soon as possible because these tumours can recur locally with a high rate and may also metastasise to bone, lymph nodes, or visceral organs. Sentinel lymph node biopsy may detect subclinical metastases originating from sweat gland carcinomas and reaching to regional lymph nodes [35]. So before initial resection, sentinel lymph node mapping and biopsy may provide useful information for guidance in early treatment [14, 35]. If there is no proven distant metastases, clinically involved lymph node regions should undergo dissection and irradiation, whereas clinically uninvolved primary lymph node regions should undergo either dissection or irradiation [13, 36]. However, based upon the high incidence of distant metastases, surgical removal of regional lymph nodes is therapeutically recommended [14, 35]. As there is no proven clear evidence of usefulness of the selective neck dissection, its role is still under debate [13, 36]. For hidradenocarcinoma, five-year postsurgical survival rate is less than 30% [11].

#### Radiotherapy

Local recurrence rates following surgery range from 10–50% [7]. Radiotherapy is usually not proposed as a first-intention therapy for cutaneous adnexal carcinomas [14]. Adjuvant radiation therapy is necessary when surgery is impossible, either because the tumour is unresectable or when a second surgical step, theoretically mandatory in case of incomplete primary surgery, is impossible or results in major local defects [14]. Radiation therapy may be given in the presence of factors of local recurrence like positive resection margins, vascular emboli, perineural invasion or nerve-sheath involvement, depth of infiltration, dermal lymphatic invasion, highly anaplastic morphology [39, 40, 41] complete resection of large tumours for nodal sterilisation, recurrent tumours, and residual lymph nodes when further surgery is not possible [7, 37]. When radiotherapy is used, high doses ranging from 50 Gy–70 Gy are recommended [14, 41]. For hidradenocarcinomas with positive margins after surgery, Harari and colleagues demonstrated complete remissions after radical external beam radiotherapy, however, the technique and dose of radiotherapy are not consensual. Harari *et al* administered 70 Gy to primary surgical beds and 50 Gy to regional lymphatic chains, using a combination of photons and electrons. In another study, two patients were administered hyperfractionated radiotherapy to minimise late normal tissue effects [41]. Because of the effects on normal tissues, both acute and long-term sequelae of external beam radiation therapy for head and neck cancer can occur. Complications are dose dependent and may vary according to the primary site. Some of the common toxicities include mucositis, xerostomia, hearing loss, trismus, and facial nerve dysfunction. Severe late toxicities include the risk of orocutaneous fistula, soft tissue necrosis, osteoradionecrosis, blindness, and second malignancies [42]. These radiotherapy-induced toxicities are less frequent and better tolerated with new techniques of radiotherapy [13]. So, radiotherapy has been used in several cases of hideroadenocarcinomass with conflicting results; in some reports it appeared to be effective, while in others, radio-resistance was observed. Regarding combined radiochemotherapy, some authors have concluded that adjuvant chemotherapy and radiotherapy have no impact on local control or survival [16].

#### Chemotherapy

Various chemotherapy regimens have also been reported [43]; first line agents included are 5- fluorouracil based regimen [7] and capecitabine (oral 5-fluorouracil) [7, 15], and second line agents included doxorubicin, platins [7] cyclophosphamide, vincristine, and bleomycin [14].

Until now, the efficiency of adjuvant chemotherapy has not been demonstrated either alone or in combination with radiation therapy [10]. Since the cellular targets are similar in hidradenocarcinoma, cancer colon, and cancer breast, and capecitabine is also recommended with positive benefits in breast and colon cancer patients, therefore, capecitabine is also useful in patients with metastatic hidradenocarcinoma [10]. Greater than 50% clinical remission was noted with this treatment, that too, with acceptable biological and clinical tolerance. This result is based on a single patient study, and such a limited objective response requires further study. Other than this, only two cases have been reported, one with elderly patient who presented with metastasis to local, regional, and distant sites such as lungs, pleura, and myocardium [44]. The patient died within a few months despite administering chemotherapy with vincristine, and bleomycin. In the second study, two children were studied with hidradenocarcinoma that had metastasised to liver, lungs, and pleura. First child was treated with cyclophosphamide and vincristine and second child was administered a combination of actinomycin D, vincristine, VM-26, dacarbazine, and doxorubicin [10]. Despite this treatment, disease progression was seen. Therefore, further studies are needed to determine the advantages of chemotherapy in terms of patient survival, to minimise or prevent disease recurrence, and for preventing metastasis. It is clear that the lack of cases remains a major obstacle to clinical trials or large studies to address the role of chemotherapeutic agents in rarely occurring hidradenocarcinoma [10]. The role of chemotherapy in sweat-gland carcinoma remains unclear [27].

#### Targeted therapy

Trastuzumab is used as an effective targeted therapy for treating various solid cancers. In the management of metastatic hidradenocarcinoma, trastuzumab also plays a role by stabilising the disease [7]. Hidradenocarcinoma and adenocarcinomas of salivary gland and breast has a histomorphologic overlap, which led to the investigators to compare ER, PR, AR, HER-2/neu status, and gross cystic disease fluid protein levels. Investigators have proved the usefulness of tamoxifen in metastatic hidradenocarcinomas with positive estrogen receptor [10]. In a case report, a patient was treated with wide local excision and regional lymph node dissection followed by external beam radiotherapy to the tumour bed and axilla. Adjuvant chemotherapy was administered with a combination of cyclophosphamide and doxorubicin. Trastuzumab was also added to the treatment regimen depending on the HER-2/neu status. The patient tolerated the therapy well and till date no signs of local recurrence have been seen [10].

#### Other treatment modalities

The role of EGFR inhibitors remains unclear in eccrine-apocrine carcinomas with protein overexpression. Targeted therapy such as Akt/PI3K/mTOR pathway inhibitors, may have a role in hidradenocarcinomas but these are in clinical testing currently [24]. Hormonal agents like antiandrogens may have a role in treating hidradenocarcinoma as these tumours express AR receptors [14, 24]. At present, there are no consolidating studies regarding the role of antiandrogen therapy. In the future follow-up clinical trial studies, it will be of paramount importance to know the projection of these results into real therapeutic response [24].

Exploitation of the molecular genetic profiling of hidradenocarcinoma may provide better insight into the identification of targeted modalities, and thus it remains a field of paramount importance [45]. In case of distant metastasis, surgery can be proposed but this possibility remains very rare. In other cases, chemotherapy is possible but the response rate is low [14].

#### Electrochemotherapy

Electrochemotherapy (ECT) is a recent treatment option for cancer involving a combination of locoregional or intravenous administration of very low doses of an antineoplastic agent (usually bleomycin or cisplatin) with electroporation of the cellular membranes. Electroporation improves penetration of chemodrugs into the cytoplasm, and results in high levels of cytotoxicity in cancer cells with fewer side effects. For all forms of solid tumours, ECT offers a safe, non-thermal, well-tolerated treatment modality suitable especially for cutaneous and subcutaneous areas. ECT acts synergistically with RT or with a combination of ECT plus RT plus systemic chemotherapy, as reported by Shil *et al* and Kranijc *et al*. Hellenic Group of Electrochemotherapy was the first to report the use of external RT combined with ECT-bleomycin in humans. So, for all types of skin tumours in the head and neck region, combined therapy of concomitant ECT and RT may be of excellent therapeutic outcome, where cosmesis and function of the healthy surrounding tissues can be safely preserved [16].

### Prognosis

Early diagnosis is critical to treatment outcome and quality of life for patients [10]. The fivc-year postsurgical survival rate is less than 30% [11]. Local recurrence rates following surgery range from 10–50% [7]. RT has conflicting results; in some reports it appeared to be effective, while in others, radio-resistance was observed. Regarding combined radiochemotherapy, some authors have concluded that adjuvant chemotherapy and radiotherapy have no impact on local control or survival [16].

### Follow-up

Patients should be regularly and closely kept in follow-up on a monthly basis. At each follow-up, the patients should be examined for any local recurrence, metastatic spread, and radiation toxicity. If clinically suspected, radiological investigations including x-ray, CT scan, and PET scan may be considered [14].

## Conclusion

Hidradenocarcinoma is a very aggressive tumour characterised by the high frequency of locoregional recurrence and notorious behaviour for distant metastases. The wide excision surgery is the mainstay of the treatment. The natural history of this rare and aggressive tumour is characterised by high rate of local recurrences. Use of external beam RT is controversial for better local control. Future trends involve chemotherapy, targeted therapy, hormonal therapy, PI3K/Akt/mTOR pathway inhibitors, EGFR inhibitors, ECT, and molecular genetic profiling, but these studies require the inclusion of a larger number of patients.

## Conflicts of interest

The authors have no conflicts of interest to declare.

## Figures and Tables

**Table 1.** Classification of cutaneous sweat gland adnexal lesions.

S. No.	Origin	Benign	Malignant
**1.**	**Eccrine**	Poroma Hidradenoma Spiradenoma Cylindroma Syringometaplasia Syringoma Syringofibroadenoma Chondroid syringoma	Porocarcinoma Hidradenocarcinoma Spiradenocarcinoma Malignant cylindroma Syringoid carcinoma Microcytic adnexal carcinoma Mucinous carcinoma Adenoid cystic carcinoma Digital papillary adenocarcinoma
**2.**	**Apocrine**	Syringocystadenoma papilliferum Hidradenoma papilliferum	Syringocystadenocarcinoma Apocrine carcinoma Extramammary Paget’s disease
**3.**	**Eccrine and apocrine (mixed origin)**	Hidrocystoma Apocrine/eccrine nevus Tubulopapillary hidradenoma	Malignant mixed tumour of skin
**4.**	**Other sweat gland carcinomas**		Eccrine ductal carcinoma Basaloid eccrine carcinoma Clear cell eccrine carcinoma Other non-specified sweat gland carcinomas

**Table 2.** TNM classification and staging system for hidradenocarcinoma.

Primary tumour (T)	
TX	Primary tumour cannot be assessed
T0	No evidence of primary tumour
Tis	Carcinoma *in situ*
T1	Tumour ≤ 2 cm in greatest dimension with < 2 high risk features†
T2	Tumour > 2 cm in greatest dimension or tumour of any size with ≥2 high risk features
T3	Tumour involving maxilla, mandible, orbit, temporal bone
T4	Tumour invading skeleton or perineural invasion in skull base

Regional lymph nodes (N)	
NX	Regional lymph node cannot be assessed
N0	No regional lymph node metastasis
N1	Metastasis in single regional ipsilateral lymph node, not ≥3 cm in greatest dimension
N2a	Metastasis in single regional ipsilateral lymph node, > 3 cm but < 6 cm
N2b	Metastasis in multiple regional ipsilateral lymph nodes, none > 6 cm
N2c	Metastasis in bilateral or contralateral regional lymph nodes none > 6 cm
N3	Metastasis in a lymph node, greater than 6 cm in greatest dimension

Distant metastasis (M)	
Mx	Distant metastasis cannot be assessed
M0	No distant metastasis
M1	Distant metastasis

Stage grouping			
Stage	T	N	M
**Stage 0**	Tis	N0	M0
**Stage 1**	T1	N0	M0
**Stage 2**	T2	N0	M0
**Stage 3**	T3	N0	M0
	T1, 2, 3	N1	M0
**Stage 4**	T1, 2, 3	N2	M0
	T4	N3	M1

† High risk features:
Depth/invasion- > 2 mm thickness, Clark > IV, perineural invasion Anatomic location-primary site – ear/non hair bearing lip Differentiation- poorly differentiated/ undifferentiated tumour

**Table 3.** Prognostic groups of sweat gland carcinomas.

S. No.	Prognosis	Risk of local recurrence	Risk of distant metastasis	Example	Surgical margin	Clinical follow- up
1.	Good	Low	Low	■ Trichilemmal carcinoma	1 cm	Every six months
2.	Intermediate	High	Low	■ Microcystic adnexal carcinoma ■ Adenoid cystic carcinoma ■ Mucinous carcinoma	2–3 cm (5 cm for microcystic adnexal carcinoma)	Every three months
3.	Poor	High	High	■ Porocarcinoma ■ Hidradenocarcinoma ■ Apocrine carcinoma ■ Sebaceous carcinoma ■ Pilomatrix carcinoma	At least 3 cm	Every month
